# Presumptive Rhino-Orbital Mucormycosis Secondary to Corticosteroid Therapy in a Diabetic Patient With COVID-19 Infection

**DOI:** 10.7759/cureus.35199

**Published:** 2023-02-19

**Authors:** Jasvinjeet K Sidhu, Wan-Hazabbah Wan Hitam, Liza Sharmini Ahmad Tajudin

**Affiliations:** 1 Department of Ophthalmology and Visual Science, School of Medical Sciences, Health Campus, Universiti Sains Malaysia, Kota Bharu, MYS; 2 Eye Clinic, Hospital Universiti Sains Malaysia, Kota Bharu, MYS

**Keywords:** corticosteroid treatment, optic perineuritis, rhino-orbital mucormycosis, post covid-19, orbital apex syndrome

## Abstract

The coronavirus disease 2019 (COVID-19) pandemic has led to the widespread use of steroids as a life-saving measure. In patients with preexisting diabetes, the therapeutic use of steroids coupled with poorly controlled sugar has led to a surge of mucormycosis. We report a rare case of orbital apex syndrome secondary to mucormycosis post-COVID-19. A 43-year-old female with poorly controlled diabetes mellitus presented with right eye complete ptosis one week post-recovery from COVID-19 infection. During COVID-19 hospitalization, she received a course of dexamethasone. The visual acuity of the right eye was 6/60. She had complete ophthalmoplegia and diplopia in all gazes. There was a positive relative afferent pupillary defect (RAPD) and reduced optic nerve function test in the right eye. MRI showed right ethmoid sinusitis with possible extension to the right orbit and the presence of right perineural optic nerve enhancement. The nasal scope revealed fungal-like thick mucopurulent discharge at the middle meatus. She was clinically diagnosed with rhino-orbital mucormycosis and was started on antifungal for six weeks. Her overall condition improved with 6/6 visual acuity and minimum residual ophthalmoplegia. In conclusion, corticosteroid treatment for COVID-19 infection in diabetic patients causes poor glycemic control and immunosuppression that can lead to secondary infections such as rhino-orbital mucormycosis.

## Introduction

As of January 2023, approximately five million people in Malaysia have been diagnosed with coronavirus disease 2019 (COVID-19), and more than 36,000 people have died because of the illness [1]. COVID-19 infection causes severe impairment in both the innate and humoral immune systems. The widespread use of steroids has been mandated as a life-saving response to COVID-19. There has been a significant increase in the number of mucormycosis cases post-COVID-19 worldwide because of the widespread use of steroids, especially in patients with preexisting poorly controlled diabetes mellitus [2].

Mucormycosis is a filamentous fungus of the family Mucoraceae. It is present in the natural environment and causes severe infection, especially in immunocompromised patients [3]. Fungal invasion happens across the nasal mucosa into the paranasal sinuses, and it invades the orbit through the medial orbital wall, subsequently involving the orbital apex and leading to orbital apex syndrome [4]. This severe complication post-COVID-19 has been reported in developing countries such as India and a few cases in western countries [5,6]. This paper describes a case of a patient with orbital apex syndrome secondary to rhino-orbital mucormycosis one week post-recovery from COVID-19 infection.

## Case presentation

A 43-year-old female, with underlying poorly controlled diabetes mellitus, presented to our center with binocular diplopia on primary gaze, partial ptosis of the right eye, and right-sided headache. She was just discharged from the ward a week before the presentation where she was admitted and treated for severe COVID-19 infection, category 4a, with superimposed bacterial lung infection requiring noninvasive ventilation. During admission, her sugar was ranging between 15 and 21 mmol/liter. She was stabilized and discharged on day 7 of treatment with a tapering dose of oral dexamethasone to complete for a total of two weeks. Upon examination, her vision was 6/6 for both eyes. There was a presence of right eye partial ptosis not covering her visual axis and diplopia on primary gaze. There was a limitation of her right extraocular movement (EOM) upon looking up. Otherwise, optic nerve function and other cranial nerve (CN) functions were intact. Her vital signs were normal; however, her sugar was 18 mmol/liter. A contrast CT scan of the brain and orbit showed no space-occupying lesion. The patient was diagnosed with isolated third cranial nerve palsy with pupil sparing secondary to uncontrolled diabetes mellitus. She was allowed to discharge home with a follow-up date within one week.

**Figure 1:**
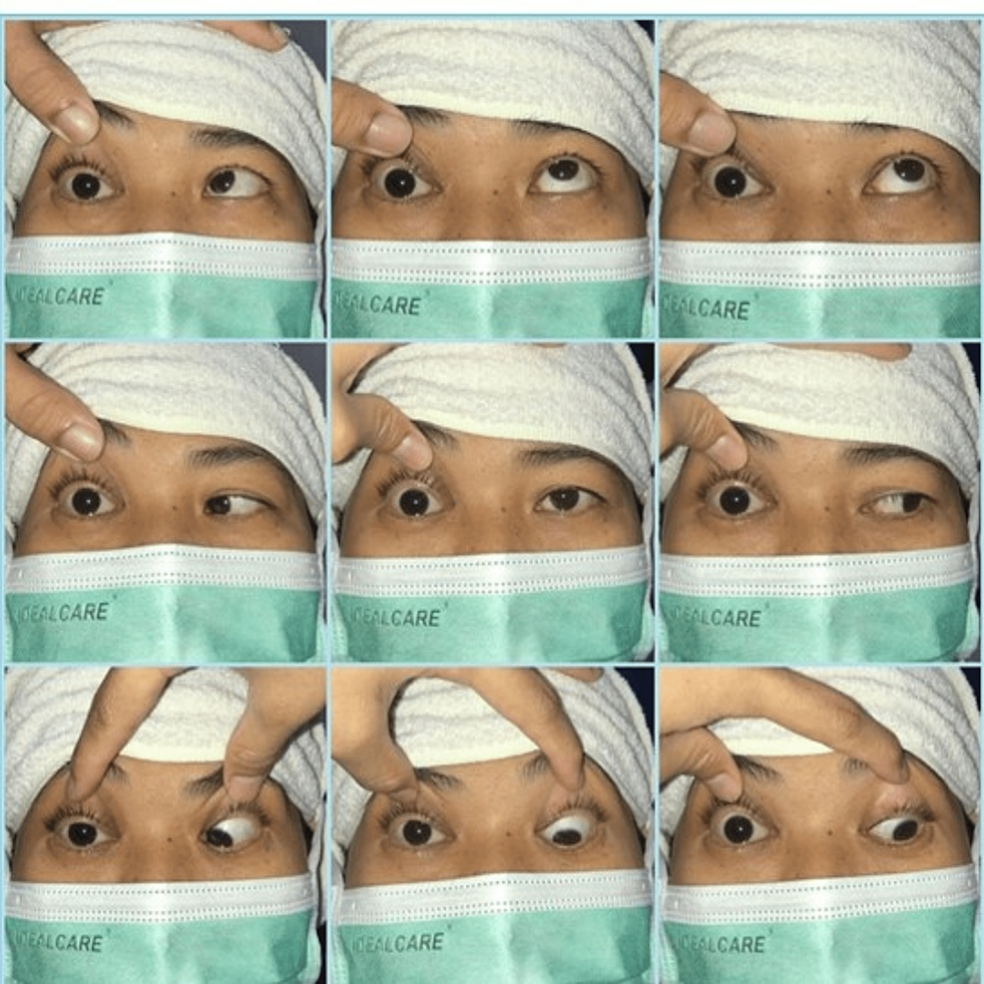
Nine cardinal gaze pictures showing complete ophthalmoplegia of the right eye secondary to orbital apex syndrome

She revisited our emergency department three days later with right eye complete ptosis and worsening right-sided headache. On examination, she had complete ophthalmoplegia of the right eye and restriction of movements in all planes (involvement of II, III, IV, and V ophthalmic division and VI cranial nerves) (Figure 1). Her right eye vision was 6/60 with impaired light brightness and red desaturation up to 20%, right eye pupil mydriasis (5 mm), and reverse relative afferent pupillary defect (RAPD) positive. Fundoscopy revealed normal findings, with an optic disc ratio of 0.3, pink, and a well-defined margin. She had poor sugar control of 19 mmol/liter. MRI of the brain showed the presence of right intraorbital collection at the orbital apex with adjacent medial temporal dural extension, the presence of right ethmoid sinusitis with extension to the right orbit, and right optic nerve enhancement. The patient was referred to the otorhinolaryngology (ORL) team, and a nasal scope was performed that revealed fungal-like thick mucopurulent discharge at the middle meatus. The nasal swab for culture and sensitivity was negative. Peripheral blood for bacterial and fungal culture also revealed no growth. No sample for histopathological examination (HPE) was taken.

**Figure 2:**
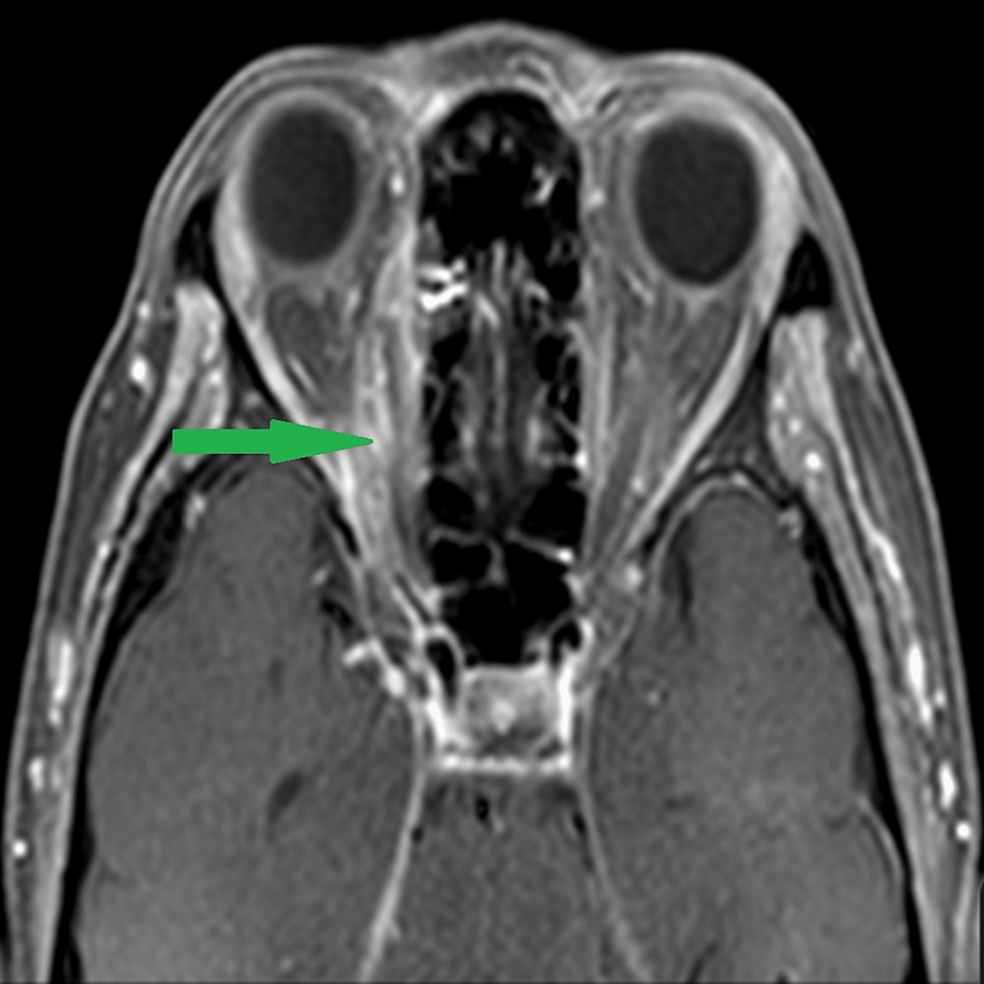
Axial cut of contrast MRI of the brain and orbit showing ill-defined lesion at the right orbital apex at the superomedial margin of the optic nerve with the presence of enhancement of adjacent intraconal and extraconal fat (green arrow) MRI: magnetic resonance imaging

**Figure 3:**
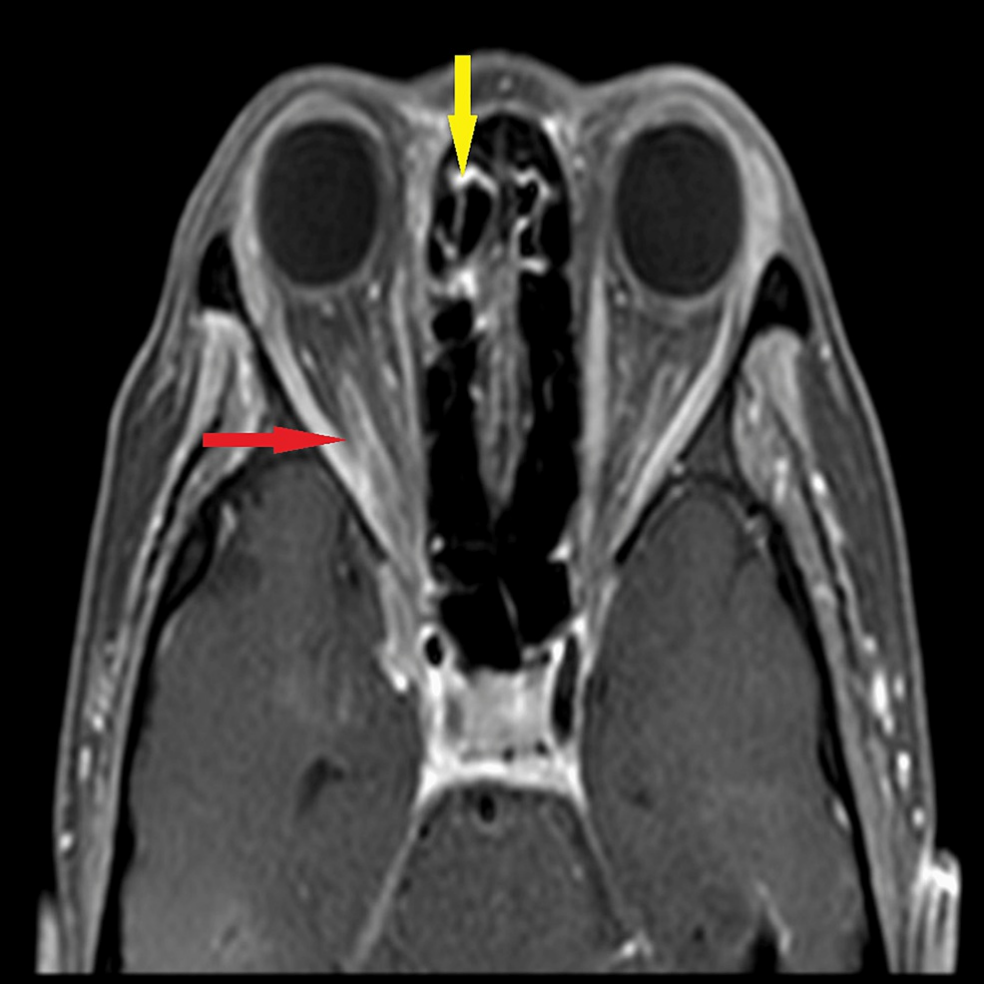
Axial cut of contrast MRI of the brain and orbit showing enhancement of the right optic nerve sheath (red arrow) and mucosal enhancement of the right ethmoid sinus (yellow arrow) MRI: magnetic resonance imaging

The diagnosis of right eye orbital apex syndrome secondary to rhino-orbital mucormycosis was made after considering the findings of the MRI with the presence of an ill-defined collection at the right orbital apex at the superomedial margin of the right optic nerve, optic nerve sheath enhancement, and mucosal enhancement of the right ethmoid sinus (Figure 2 and Figure 3). A multidisciplinary team discussion between the ophthalmologist, ORL surgeon, radiologist, and infectious disease specialist decided to empirically treat the patient without repeating nasal scope for HPE. The patient was started on intravenous amphotericin B. On day 7 of treatment, she developed hyperkalemia, and the antifungal treatment was revised to intravenous voriconazole. Upon the completion of intravenous antifungal (amphotericin and voriconazole) for a total of 14 days, her condition improved. Her right eye vision improved to 6/9, and her optic nerve function was equal between both eyes. However, RAPD remains positive in the right eye with complete ptosis and ophthalmoplegia.

The patient was discharged with oral voriconazole to complete for six weeks. Upon review at the outpatient clinic after completing treatment, the patient’s right eye vision was 6/9, with full extraocular movement in all planes with improvement of ptosis. However, RAPD remains positive. Repeated MRI six weeks post-antifungal treatment showed that the ill-defined lesion at the superomedial aspect of the right orbit was smaller in size. The optic nerve appears unremarkable with normal signal intensity. The patient was under regular follow-up, and her condition remains stable.

## Discussion

Severe COVID-19 infection is frequently linked with the onset of secondary infections, with fungal infection being 10 times more common [7]. Mucormycosis has emerged as a potentially lethal complication of this disease. Recently, with the surge of COVID-19 cases, there has been an increase in the number of mucormycosis cases with the rhino-orbital form predominating [5]. A multicenter study conducted in India reported a 2.1-fold increase in the number of cases associated with mucormycosis during the pandemic compared to the previous year [8]. In Malaysia, this is the first case reported post-COVID-19 in the northeast region of the country.

Mucormycosis is a serious and highly aggressive fungal infection known to have high morbidity and mortality rate if not treated promptly. It is an angioinvasive infection that can cause gangrene and tissue infarction [7]. The diagnosis of mucormycosis is challenging if indicators such as thrombosis and necrotic tissue are absent or are late in presentation [9]. The major risk factors associated with this infection include diabetes, corticosteroid therapy, hematological and solid organ malignancies, transplant recipients, and neutropenia [10]. The risk factors associated with healthcare outbreaks include prolonged oxygen support, poor air filtration, and non-sterile medical instruments [5,7].

The main risk factors associated with mucormycosis in COVID-19 patients include hypoxia, which creates an ideal environment for the proliferation of fungi, hyperglycemia secondary to underlying diabetes mellitus, prolonged use of high-dose corticosteroids, and a weakened immune system [11]. The use of corticosteroids leads to both immunosuppression and immune dysregulation, besides contributing to hyperglycemia [12]. The immunocompromised state of the patient together with high fungal spore counts in the hospital environment creates an ideal setting for mold infections [7]. The patient we reported was treated with corticosteroids during hospitalization for COVID-19 infection, and she also has poorly controlled underlying diabetes mellitus, worsening due to the use of corticosteroids. The combination of these factors has rendered our patient susceptible to mucormycosis infection.

The orbital apex region is susceptible to infection, as the infection spreads from the paranasal sinuses. The involvement of the orbital apex is characterized by vision loss from the involvement of the optic nerve, ophthalmoplegia with the involvement of the oculomotor nerves (namely, CN III, IV, and VI), and the involvement of the first division of the trigeminal nerve (V1) [4]. A study conducted by Srivastava et al. reported that the prevalence of orbital apex involvement is as high as 34.8% in COVID-19-associated mucormycosis (CAM). Among these patients, 74.2% had a vision of light perception that subsequently led to permanent loss of vision [2]. In diabetics, orbital apex syndrome secondary to mucormycosis may initially present as a partial ophthalmoplegia and is felt to represent microvascular disease, especially when there are minimal sinus findings by CT scan [9]. Similarly, our patient initially presented with the complaint of right eye partial ptosis and upward gaze restrictions. She was diagnosed with medical third cranial nerve palsy secondary to poorly controlled diabetes mellitus and was allowed discharge. However, her condition deteriorated within three days from the initial presentation, and she developed orbital apex syndrome secondary to mucormycosis.

Blood tests are relatively preliminary in the diagnosis of mucormycosis, and diagnosis mostly relies on histopathological evidence. Even in disseminated disease, blood cultures are mostly negative [7]. It is difficult to grow the mucormycosis fungi in culture media. Honavar reported that about 50% of the samples sent are culture-negative [13]. In histological evidence, Mucorales have quite a distinct appearance. It has irregular non-septate hyphae that branch at right angles [7]. For our patient, there was no histopathological evidence of mucormycosis, and the nasal sample and swab sent did not grow fungal hyphae. Peripheral blood was sent for bacterial and fungal culture, and sensitivity was reported as no growth.

Radiological imaging is of paramount importance. CT and MRI findings in the early stage, seen as variable density and intensity of sinuses, mucosal thickening, and intra-sinus contents, are nonspecific and resemble the more often encountered simple rhinosinusitis. Several radiological findings are useful in predicting the diagnosis of mucormycosis presence of bone erosions, picked up on a plain CT scan [14]. In a study by Mangal et al., he reported that the most frequent finding on MRI associated with mucormycosis is the presence of mucosal thickening of the sinuses, with hypertrophy and irregular mucosal thickening of the nasal cavity. Other findings seen in the orbit include ill-defined fat stranding involving extraconal and intraconal space, enlargement of the optic nerve seen on MRI T2/short tau inversion recovery (STIR) hyperintense signal, diffusion restriction, and enhancement. Perineuritis was seen as a thickening and prominent enhancement of the optic nerve sheath [15]. In our study, there were similar findings such as mucosal thickening of ethmoid sinuses, enhancement of intraconal and extraconal fat, and enhancement of optic nerve sheath.

Orbital mucormycosis requires early and aggressive management. Optimal therapy requires a multidisciplinary discussion between neurologists, otorhinolaryngologists, ophthalmologists, and infectious disease specialists. The ideal management is surgical debridement under the cover of amphotericin B. In a study conducted in India, patients treated with intravenous amphotericin B with surgical debridement of necrotic tissue showed a lower mortality rate of 19%-44% in comparison with those treated with intravenous amphotericin B alone, with a mortality rate of 50%-61% [16]. In our case, our patient responded well to intravenous amphotericin and voriconazole without requiring any surgical intervention. Her condition improved upon completing the antifungal treatment for a total of six weeks, although there was residual impairment of cranial nerve II.

This case illustrates the need for a regular and attentive follow-up of patients with risk factors during and after severe COVID-19 infection. The diagnosis of orbital apex syndrome secondary to rhino-orbital mucormycosis was made in this case because of the highly localizing symptoms, combined with the patient’s risk factors, and MRI findings, despite negative culture yield. Mucormycosis is a very rare and destructive disease, and early diagnosis is crucial for a better outcome. A multidisciplinary team approach is important for timely treatment and intervention to reduce morbidity and mortality. When any sinus disease with orbital apex syndrome is observed in a diabetic or immunocompromised patient by CT or MRI, fungal etiology should be suspected. MRI is an effective modality in diagnosing orbital apex syndrome, with biopsy being the most reliable.

## Conclusions

As we recover from the COVID-19 pandemic, we are faced with many complications of the disease and the treatment instilled. Rhino-orbital mucormycosis is a devastating complication of corticosteroids used for the treatment of COVID-19 infection, especially in diabetics.
